# A Systematic Review of *In Vitro* Activity of Medicinal Plants from Sub-Saharan Africa against *Campylobacter* spp.

**DOI:** 10.1155/2020/9485364

**Published:** 2020-05-15

**Authors:** Delfina Fernandes Hlashwayo, Filomena Barbosa, Sílvia Langa, Betuel Sigaúque, Custódio Gabriel Bila

**Affiliations:** ^1^Departamento de Ciências Biológicas, Faculdade de Ciências, Universidade Eduardo Mondlane, Avenida Julius Nyerere nr 3453, Campus Principal, Maputo, Mozambique; ^2^Faculdade de Veterinária, Universidade Eduardo Mondlane, Avenida de Moçambique, Km 1.5, Maputo, Mozambique; ^3^Centro de Investigação em Saúde de Manhiça, Maputo, Mozambique

## Abstract

**Introduction:**

*Campylobacter* spp. are zoonotic bacteria that cause gastroenteritis in humans and may cause extraintestinal infections such as Guillain-Barré syndrome, reactive arthritis, and bacteremia. Resistance to antibiotics is an increasing concern in the Sub-Saharan Africa; thus, search for alternatives such as plant-based active ingredients is important in order to develop new drugs.

**Objectives:**

To present a systematic review of *in vitro* and *in vivo* studies of the antibacterial activity of medicinal plants from Sub-Saharan Africa against *Campylobacter* spp. *Methodology*. Studies published until March 2020 on medicinal plants used against *Campylobacter* spp. from each country of Sub-Saharan Africa were searched on PubMed, Science Direct, AJOL, and Google Scholar. Articles were selected based on the existence of information regarding *in vitro* and *in vivo* activity of medicinal plants against *Campylobacter* spp.

**Results:**

A total of 47 medicinal plants belonging to 28 families were studied for *in vitro* activity against *Campylobacter* spp. No plant was studied *in vivo.* Plants from Fabaceae family were the most commonly studied. The plants with the strongest antimicrobial activities were *Cryptolepis sanguinolenta* and *Terminalia macroptera*. The root extracts from these plants were effective, and both had a minimal inhibitory concentration (MIC) of 25 *μ*g/ml. Seven pure compounds were isolated and analyzed for activity against *Campylobacter* spp. The compound cryptolepine from *C. sanguinolenta* was the most effective with MIC values ranging between 6.25 and 25 *μ*g/ml.

**Conclusion:**

Several native plants from the Sub-Saharan Africa region were studied for *in vitro* activity against *Campylobacter* spp. Some plants seemed very effective against the bacteria. Chemical compounds from three plants have been isolated and analyzed, but further studies are needed in order to produce new and effective drugs.

## 1. Introduction

*Campylobacter* spp. are a group of zoonotic bacteria among the leading causes of human bacterial gastroenteritis. They account for 5% to 14% of all diarrheal diseases in the world [1, 2]. In humans, *Campylobacter jejuni* and less often *C. coli* cause watery or bloody diarrhea, fever, abdominal cramps, and vomiting. Enteritis caused by these bacteria is sporadic and self-limiting. However, complications such as bacteremia, hepatitis, pancreatitis, lung infections, brain abscesses, meningitis, and reactive arthritis may occur, and immunocompromised individuals are at higher risk [3–5].

*Campylobacter* spp. are gram-negative, mobile, and non-spore-forming bacteria. They colonize the gastrointestinal tract of various host species as commensals such as birds and ruminants, including cattle, sheep, and goats [5, 6]. The bacteria can also cause diarrhea in these animals [7]. Nevertheless, bacteria from this genus are the cause of miscarriages and infertility in ruminants [8].

The widespread of *Campylobacter* spp. in Sub-Saharan Africa may be explained by poor hygiene and lack of sanitation. Additionally, direct contact between people and animals and ingestion of contaminated food and water are some of the sources of *Campylobacter* spp. contamination [9].

Antibiotics may be considered for treatment of *Campylobacter* spp. infections in severe cases [1]. The commonly used drugs to treat campylobacteriosis in humans are erythromycin, fluoroquinolones, or tetracycline [10]. Although this antimicrobial treatment is generally not necessary, misuse of antibiotics is common in Sub-Saharan Africa and this is leading to increasing antibiotic resistance [10–13].

Recently published systematic reviews reported antibiotic resistance of *Campylobacter* spp. isolates from Sub-Saharan Africa to drugs used for clinical treatment of campylobacteriosis in severe cases, such as erythromycin, ampicillin, tetracycline, and ciprofloxacin [7, 13]. This drug resistance is a major global public health concern that leads to treatment failure.

*Campylobacter* spp. are on the World Health Organization (WHO) list of global priority pathogens for research and development of new antibiotics [14]. Several antibiotics are no longer effective in the clinical treatment of campylobacteriosis, so new antibiotics and novel treatment schemes are needed [15]. In this context, medicinal plants are promising in isolating candidate molecules for new drugs [16], since phytochemicals are a major source of bioactive compounds with potent antimicrobial activities [17].

Medicinal plants have been used for a long time for treatment of several diseases. This knowledge has been passed through generations [18]. About 88% of the population in the African region was reported to be using traditional and complementary medicines (T&CM) for healthcare as of 2018 [19], which includes plant-based therapies.

Natural products, including the ones from plants, have been the main source of drugs, including antimicrobial agents. Moreover, currently many medicinal plant extracts are used as prescription drugs in developed countries [18].

One of the objectives of the WHO Traditional Medicine Strategy 2014–2023 is to promote universal health coverage by integrating T&CM services into health service delivery and self-healthcare by capitalizing on their potential contribution to improve health services and health outcomes [20]. This systematic review provides useful data on effective plants for treatment of campylobacteriosis in severe cases, as well as candidate compounds for new and more effective antibiotics, contributing to the above described WHO objective.

Ethnobotanical information from Sub-Saharan Africa regarding the plants used for treatment of diarrhea is available for many countries. As a result of these studies, *in vitro* studies were conducted in order to analyze the antimicrobial effect of these plants or phytochemical compounds against *Campylobacter* spp.

The antimicrobial activity of a plant extract or of an antibiotic can be determined by disk diffusion method, where the active compound is diffused into an agar plate of microorganisms by a disk or by wells [21]. However, the best way to express the antibacterial activity of a compound or an extract is the minimal inhibitory concentration (MIC). It is the lowest concentration of a substance that inhibits the growth of a microbe by broth microdilution method [21]. For this reason, the plants extracts and compounds with strongest antibacterial activities were defined by their MIC values in this review.

It is important that Sub-Saharan African countries start to use local resources for the development of drugs. Therefore, this work intends to analyze the *in vitro* and *in vivo* studies of medicinal plants from Sub-Saharan Africa against *Campylobacter* spp. in order to identify effective extracts and chemical compounds against the bacteria that may be the subject of further studies for development of new drugs. To our knowledge, there is no systematized data for *in vitro* or *in vivo* activity of medicinal plants against this pathogen. Thus, this review is a pilot research that may direct future research to identify drug candidate molecules in Sub-Saharan African flora.

## 2. Material and Methods

### 2.1. Search Strategy

A systematic review was conducted according to PRISMA guidelines to find data available for medicinal plants from each country of Sub-Saharan Africa used against *Campylobacter* spp. The following keywords were used: campylobacteriosis, *Campylobacter*, and medicinal plant, along with the names of each country from Sub-Saharan Africa. The UN macro-geographical definition of Africa was used to define the geographical boundaries of this review (https://unstats.un.org/unsd/methodology/m49/). The detailed search strategy is found in Supplementary Material 1.

PubMed, Google Scholar, African Journals Online, and Science Direct were searched for studies published up to 12 March 2020 without language and time restrictions. Studies evaluating the *in vitro* activity of medicinal plants against the bacteria were included. No *in vivo* studies were found.

Titles and abstracts were screened for location and correlation with the research objectives. Full versions of potentially relevant articles were obtained to assess eligibility. These were then independently evaluated for inclusion.

### 2.2. Data Extraction

Data regarding scientific names of plants, botanical family, plant parts, country of plant origin, type of extracts, isolated compounds, inhibition diameter in millimeters (mm), MIC, and minimal bactericidal concentration (MBC) in *μ*g/ml were collected independently from each publication and captured using a standardized Word document form. The scientific names of the plants were checked with http://www.theplantlist.org. Botanical families follow APG IV system [22].

## 3. Results

### 3.1. Database Search Results

**Figure 1:**
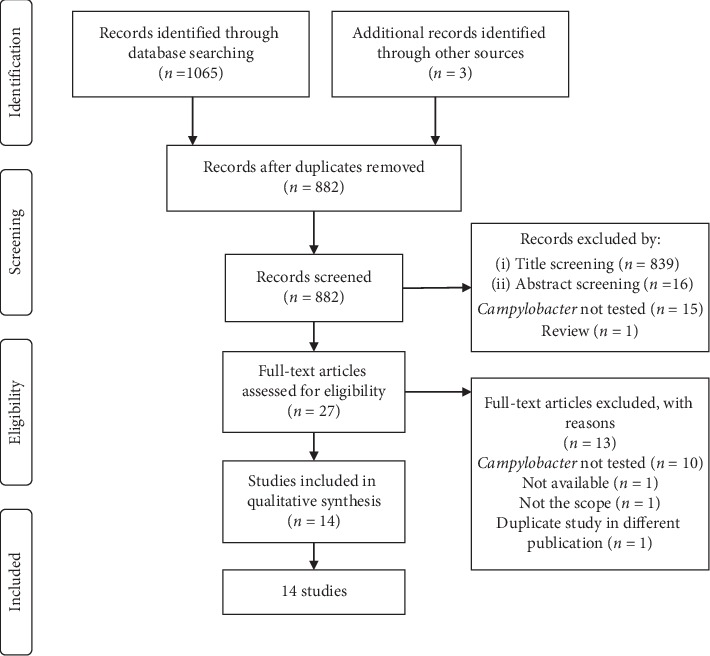
PRISMA flow diagram of study selection.

**Figure 2:**
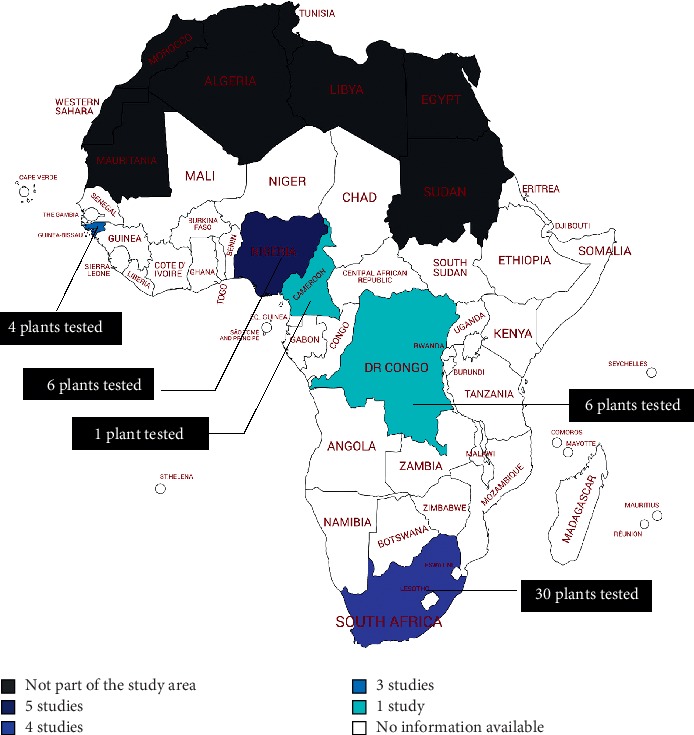
Distribution and number of included studies by country of plant origin. Map made through https://mapchart.net/africa.html.

**Figure 3:**
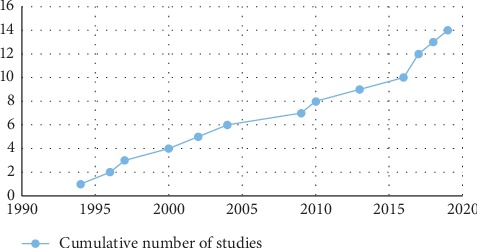
Distribution of studies by year of publication.

A total of 1065 articles were found from the initial database search, and three were found through other sources (Figure 1). After removing the duplicates (*n* = 186), 855 studies were excluded based on title and abstract. Twenty-seven full-text articles were assessed for eligibility, from which 13 were excluded. A total of 14 studies about the *in vitro* antibacterial activity of medicinal plants from Sub-Saharan Africa against *Campylobacter* spp. were found [23–36]. The majority of studies were from Nigeria (5 studies), South Africa (4), and Guinea-Bissau (3). Other countries included Democratic Republic of Congo (1) and Cameroon (1) (Figure 2). However, South Africa tested the highest number of plants (*n* = 30), while Cameroon had only one plant tested. The included articles were published between 1994 and 2019 (Figure 3).

### 3.2. Results of the *In Vitro* Studies

A total of 47 medicinal plants from Sub-Saharan Africa were studied for *in vitro* activity against *Campylobacter* spp. These plants belong to 28 families. Plants from Fabaceae family were the most common with 11 species. Other botanical families were Menispermaceae and Combretaceae (each with 3 species); Annonaceae, Apocynaceae, Asteraceae, Moraceae, and Vitaceae (each with 2 species); Anacardiaceae, Apiaceae, Celastraceae, Connaraceae, Cucurbitaceae, Euphorbiaceae, Gunneraceae, Hypoxidaceae, Iridaceae, Lamiaceae, Loranthaceae, Malvaceae, Myrtaceae, Olacaceae, Phyllanthaceae, Rubiaceae, Sapotaceae, Simaroubaceae, Urticaceae, and Verbenaceae (each with 1 specie).

Two plants, namely, *Cryptolepis sanguinolenta* and *Peltophorum africanum*, were analyzed in more than one research.

**Figure 4:**
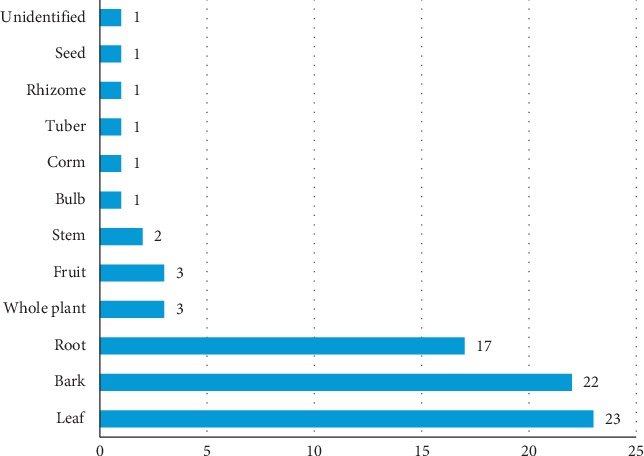
Frequency of analyzed plant parts.

The most analyzed plant parts were leaves, bark (from stem and root), and roots as shown in Figure 4.

**Figure 5:**
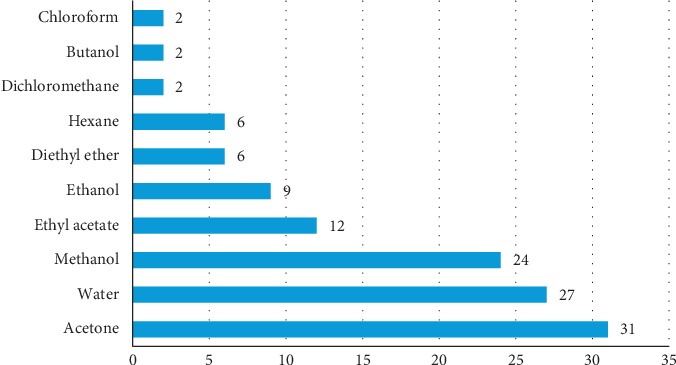
Frequency solvents used for plant extracts.

Acetone, water, and methanol were the most used solvents for plant extract preparation, while chloroform, butanol, and dichloromethane were less used (Figure 5).

Nine studies tested the antimicrobial activity for *Campylobacter* spp. through the MIC [23, 24, 26, 29–31, 33–35], while five studies tested only through disk diffusion method [25, 27, 28, 32, 36].  Table 1 provides a summary of the studied plant extracts, plant parts, isolated compounds, diameter of inhibition, MIC, and MBC of the tested plants.

Most of the plants extracts were studied for activity against *C. jejuni* (*n* = 27), followed by *Campylobacter* spp. (*n* = 20), *C. coli* (*n* = 11), *C. lari* (*n* = 2), and *C. fetus* (*n* = 1).

The plants with the greatest antimicrobial activities were *Cryptolepis sanguinolenta* and *Terminalia macroptera*, in which both ethanolic extracts of the roots presented MIC = 25 *μ*g/ml against *C. jejuni* and *Campylobacter* spp., respectively. The other plants with high antimicrobial activities were *Combretum woodii* (MIC = 40 for *C. jejuni*), *Albertisia villosa* (MIC = 62.5 for *C. jejuni* and *C. coli*), and *Lippia javanica* and *Pterocarpus angolensis* (both with a MIC = 90 for *Campylobacter* spp.).

A total of seven pure compounds were isolated from 3 plants and tested against *Campylobacter* spp. Cryptolepine, an alkaloid isolated from the ethanolic extract of *C. sanguinolenta* root, was the most effective compound with MIC ranging between 6.25 and 25 *μ*g/ml for *C. jejuni* and *C. coli* [30]. Methyl gallate from the ethyl acetate extract of *Searsia chirindensis* leaf presented a MIC of 60 *μ*g/ml against *C. jejuni* and was very effective as well [34]. Other four compounds were isolated from this plant extract, namely, myricetin-3-O-arabinopyranoside, myricetin-3-O-rhamnoside, kaempferol-3-O-rhamnoside, and quercetin-3-O-arabinofuranoside with MICs ranging between 130 and 250 *μ*g/ml [34]. However, friedelan-3-one, a terpenoid isolated from the ethyl acetate extract of *Pterocarpus santalinoides* leaf, was not effective against *C. jejuni* [32].

No *in vivo* studies of plants from the Sub-Saharan African region were found.

## 4. Discussion

This study had the aim of identifying the *in vitro* and *in vivo* activities of medicinal plants from Sub-Saharan Africa against *Campylobacter* spp. We found out that several plants were analyzed for *in vitro* activity. These results show that attention has been paid to the search for new plant-based antibiotic alternatives in the region, although no *in vivo* studies were performed to date.

A high number of plants (41/47) were tested by the MIC alone or in addition to the inhibition zone through the disk diffusion method. The disk diffusion method does not provide sufficient detail about the concentration of the extract that inhibits bacterial growth. Moreover, many factors can influence the results in such method, such as concentration of the compound in the test solution; volume of the test solution; density of the inoculum; duration and temperature of the diffusion phase before incubation; and thickness and composition of the medium, as well as incubation temperature [21]. Thus, we emphasize the importance of conducting research on the MIC values for the plant extracts and isolated compounds through broth microdilution.

Acetone, water, and methanol were the most used solvents, probably because they allow the extraction of a wide range of active principles and are not toxic. It has also been found that, in addition to the plant type, the solvent used for extraction interferes with antibacterial activity [26], most likely because it influences the isolation of chemical compounds [37]. Thus, it is interesting to study various types of solvents in order to have more accurate results.

Leaves, bark, and roots were the most studied plants parts, and this is certainly related to the ethnobotanical knowledge. In traditional African medicine, these plant parts are mostly used for the production of many remedies, including diarrhea remedies.

*Cryptolepis sanguinolenta* (Apocynaceae) was the plant with the highest antibiotic activity expressed in the lower MIC. The alkaloid cryptolepine was isolated from this plant. The root of the plant is used in traditional medicine in West Africa for the treatment of various infectious diseases including diarrhea, mostly through decoction [30, 38]. Additionally, the plant is traditionally used in the continent to treat fever, upper respiratory infections, urinary tract infections, septicemia, respiratory diseases, other enteric diseases, insomnia, amoebiasis, hypertension, inflammation, pyrexia, malaria, diabetes, stomach and intestinal disorders, tuberculosis, hepatitis, and wounds [38].

In the *in vitro* study, cryptolepine activity was higher than that of prescribed antibiotics as co-trimoxazole and sulfamethoxazole. Moreover, *Campylobacter* spp. susceptibility to cryptolepine was equal to ampicillin [30]. This is an interesting and important finding that proves that this compound has greater activity and is similar to antibiotics used in clinical practice. Thus, it has great potential as a precursor to a new effective drug. Cryptolepine has also antibacterial activity against other bacteria such as *Staphylococcus aureus*, probably because it causes morphological changes, cellular breakdown, and DNA intercalating and inhibits topoisomerase II [38]. Probably this mechanism of action is similar to what happens in *Campylobacter* spp.

*Terminalia macroptera* (Combretaceae), the second plant with the highest antibacterial activity (MIC = 25 *μ*g/ml of ethanolic root extract), is also used in West Africa for treatment of infectious diseases such as hepatitis, cough, tuberculosis, diarrhea, dysentery, fever, and malaria [23, 39]. The most used parts are roots, stem bark, and leaves, although the fruits are also used. The most common preparation method to treat diarrhea is a root decoction [23, 39].

The activity of the ethanolic extract of *T. macroptera* root against *Campylobacter* spp. was similar to the antibiotic co-trimoxazole and higher than sulfamethoxazole, although smaller than other clinically used antibiotics [23]. These data are also very important and demonstrate the great antibiotic potential of this plant. The investigators have identified that a class of polyphenols called ellagitannins are the major compounds in the extract and active fractions [23]. Additionally, the plant was found to have flavonoids, triterpenoids, and other phenolic compounds [39]. Unlike *C. sanguinolenta*, the active compounds of this plant have not been analyzed for antimicrobial activity against *Campylobacter* spp. However, the available study validates its use in the treatment of diarrhea caused by *Campylobacter* spp.

It is interesting that another plant of the Combretaceae family, *Combretum woodii*, was the third with greater antibacterial activity against *Campylobacter* spp. (MIC = 40 *μ*g/ml of the acetone and ethanolic extract of leaves). Nevertheless, unlike other species from that family, traditional medicinal use of *C. woodii* has not been reported in the literature.

At least twenty-four *Combretum* species are used in African traditional medicine to treat ailments and diseases such as scorpion and snake bites, mental and heart problems, as well as fever and microbial infections, including diarrhea [40]. *Combretum* species are prepared as hot water decoctions or cold water extracts or mixed with food, such as maize porridge. Some decoctions have proved to be as effective as alcoholic and acetone extracts. Antimicrobially active compounds isolated from *Combretum* spp. are combretastatins, acidic tetracyclic and pentacyclic triterpenes/triterpenoids, ellagitannins, phenanthrenes, flavonoids, saponins, and cycloartane glycosides [26, 40].

For the first time in 2005, the active compound present in the highest concentration in the leaves of *C. woodii* was isolated, so called Combretastatin B5 (2′,3′,4-trihydroxy-3,5,4′-trimethoxybibenzyl). This compound was very effective against *S. aureus*, *Pseudomonas aeruginosa*, and *Enterococcus faecalis* (MIC of 16, 125, and 125 *μ*g/ml, respectively), with the exception of *Escherichia coli* (>250 *μ*g/ml). It is a stilbene, a bibenzylic compound with a potent antibacterial activity, which was greater or similar to that of ampicillin and chloramphenicol against the bacteria tested, except for *E. coli* [41]. For *T. macroptera*, the active compound of this plant has not been tested against *Campylobacter* spp. However, this compound is presumed to have even greater antimicrobial activity than the extract, and this may explain the traditional use of *Combretum* species in the treatment of diarrhea caused by *Campylobacter* spp.

Another plant with a low MIC (62.5 *μ*g/ml) of the methanol and aqueous extracts was *Albertisia villosa* (Menispermaceae). This plant is native to DRC, where root decoction is ingested to treat diarrhea and dysentery. Therefore, the traditional use of the plant against *C. jejuni* and *C. coli* was validated. This plant is also promising in the discovery of new antibiotics [29]. A preliminary phytochemical testing detected alkaloids and saponins in the root of the plant [29]. Years later, bisbenzylisoquinoline alkaloids were isolated from the root bark, namely, cycleanine, cocsoline, and N-desmethylcycleanine. Cycleanine was the most abundant (85%) of all identified alkaloids. Through agar well diffusion, this compound was very effective against bacteria such as *Bacillus subtilis*, *Corynebacterium diphtheriae*, *Klebsiella pneumoniae*, *P. aeruginosa*, *Salmonella typhi*, and *Streptococcus pyogenes* and human pathogenic fungi such as *Trichophyton longiformis*, *Candida albicans*, and *Aspergillus flavus* [42].

The ethanolic and acetone extracts of the leaves of *Lippia javanica* and the ethyl acetate extract of the bark of *Pterocarpus angolensis* both had MICs of 90 *μ*g/ml for *Campylobacter* spp. These were described for the first time as effective against *Campylobacter* spp. in 2009 [33]. These plants are used in the Venda region of South Africa for treatment of several diseases. *L. javanica* leaves are used for treatment of asthma, malaria, and diarrhea. A decoction or infusion of root or leaves is taken orally to treat diarrhea [43]. Other traditional medicinal uses of the plant in Africa are to treat bronchitis, chest pains, wounds, fever/malaria, cough, and colds, as well as to repel mosquitoes [43].

*L. javanica* naturally occurs in central, eastern, and southern Africa. Alkaloids, amino acids, flavonoids, iridoids, and triterpenes have been identified from the plant [43]. The antibacterial properties of *L. javanica* can be attributed to phenolic compounds such as apigenin 7, which was highly active against *Vibrio cholera*, *E. faecalis*, *S. typhi*, *Proteus mirabilis*, and *P. aeruginosa* [43]. Therefore, these results give credence to the use of the species' infusions against bacterial infections including campylobacteriosis.

*P. angolensis* bark is used for treatment of wounds, malaria, gonorrhea, headaches, stomach aches, diarrhea, mouth sores, and rashes in Venda, South Africa [33, 44]. Tannins and saponins were the identified classes of compounds in a preliminary phytochemical screening of stem bark [45]. The water and methanol extracts of the bark are very effective against *S. aureus*, *Streptococcus agalactiae*, and *Candida krusei*, while the dichloromethane extract is effective against *S. agalactiae* and *C. krusei*. [45]. Four tannins were isolated from an ethanolic extract of the stem bark, namely, (-)-epicatechin, epicatechin-3-O-gallate, epicatechin (4b–8)-epicatechin (B2), and a hexamer of epicatechin. These compounds were effective against the following bacteria: *S. aureus*, *S. typhi*, *Micrococcus kristinae*, and *Acinetobacter calcoaceticus* [44].

Regardless of the positive results for antibacterial activity against *Campylobacter* spp., the active principles of *A. villosa*, *L. javanica*, and *P. angolensis* were also not tested against the bacteria.

*Searsia chirindensis* was one of the plants in which active principles were isolated and tested in South Africa. This plant is used in the South African traditional medicine to treat many diseases, one of which is diarrhea. From the five tested compounds isolated from the leaves of the plant, methyl gallate, a phenolic compound, presented the highest antimicrobial activity with a MIC of 60 *μ*g/ml for *C. jejuni* [34]. Methyl gallate was the second compound in addition to cryptolepine, with greater antibacterial activity against *Campylobacter* spp. in Sub-Saharan Africa. The other four tested compounds were flavonol glycosides that also exhibited a good antimicrobial activity ranging between 130 and 250 *μ*g/ml. This provides credence to the ethnomedicinal use of *S. chirindensis* to treat diarrhea, as well as describing chemical substances that may be precursors of new drugs.

Although active principles of antidiarrheal medicinal plants from Sub-Saharan Africa had good results against *Campylobacter* spp., other compounds such as friedelan-3-one, a terpene from the leaves of *Pterocarpus santalinoides*, had no activity against the bacteria. However, the compound had activity against other bacteria such as *Helicobacter pylori* that is in the same family as *Campylobacter* spp., as well as *E. coli*, an enteric bacterium [32].

A study in South Africa reported that, among many bacteria tested on a total of 11 medicinal plants extracts, *C. jejuni* was the most resistant [35], probably due to its biochemical defense mechanisms. Some of the mechanisms are the efflux pumps that remove antibiotics from the cytosol of the bacteria [46]. Other biochemical defense mechanisms include decreasing outer membrane permeability and alterations in the membrane structure or in porin proteins [15]. This fact is important and emphasizes the truth that few plants with considerable antibacterial activity against *Campylobacter* spp. have been found. In this context, plants with low MIC values are promising in the development of new drugs or in the natural treatment of diarrhea caused by these bacteria.

Other plants in which the extracts also had a high antibacterial activity (MIC below 200 *μ*g/ml) were *Zornia milneana* (whole plant), *Syzygium cordatum* (bark), *Rourea obliquifoliolata* (leaf), and *Rhoicissus tridentata* (fruit). All of these plants are used in the African traditional medicine to treat diarrhea, as well as other infectious diseases [29, 33]. However, none of them had their active compounds analyzed. These, as well as the most effective ones, can be considered as priorities for future studies aiming at isolation and analysis of their chemical compounds.

Ethnobotanical information from Sub-Saharan Africa regarding the plants used for treatment of diarrhea should be compiled in order to develop more *in vitro* and *in vivo* studies on the effects of the most cited plants against *Campylobacter* spp. There is also a need to conduct research in other countries given that the flora in this region is rich in medicinal plants that have enormous therapeutic potential.

This review has also found out that few active compounds have been isolated and analyzed for activity against *Campylobacter* spp. This isolation and pharmacological analysis should be done for plants with low MIC levels in their extracts. In this work we identified such plants as *Terminalia macroptera*, *Combretum woodii*, *Albertisia villosa*, *Lippia javanica*, and *Pterocarpus angolensis*.

Despite the isolation of pure chemical products or compounds from medicinal plants with high antibacterial activity, the other main challenge now is also to elucidate the biological mechanisms of the isolated compounds and to perform pharmacological studies. These studies would include the following: *in vitro* and *in vivo* efficacy, bactericidal or bacteriostatic activity, rate of resistance, bioavailability, *in vivo* pharmacokinetic studies, stability of the compounds in formulation, spectrum of antibacterial activity, treatment duration, and route of administration. In the absence of serious liabilities, the candidate drug might be declared for entry into a preclinical development program [47], and clinical trials could be further conducted according to the approved standards [18].

## 5. Conclusions

This review demonstrated that a total of 47 plants have been studied for *in vitro* activity against *Campylobacter* spp. in Sub-Saharan Africa. The number of tested plants is low when compared to the region's large flora. However, the region has promising plants with antibacterial activity against the bacteria.

Some plants, such as *Cryptolepis sanguinolenta* and *Terminalia macroptera* are the most effective with *in vitro* activity against *Campylobacter* spp. Besides these, *Combretum woodii*, *Albertisia villosa*, *Lippia javanica*, and *Pterocarpus angolensis* have also a strong activity against the bacteria. Of those plants with strong activity, only *C. sanguinolenta* had its active compounds isolated and tested against *Campylobacter* spp. These data demonstrate the need to isolate and test compounds from other plants as precursor steps in the discovery of novel candidate drugs.

Isolated chemical compounds such as cryptolepine and methyl gallate from *C. sanguinolenta* and *Searsia chirindensis*, respectively, have a strong antimicrobial activity. Given that *Campylobacter* spp. are rapidly gaining resistance to drugs currently in use, these compounds will need to be tested in further pharmacological studies in order to better understand the mechanisms of action and to test *in vivo*. This testing may lead to the development of new and more efficient antibiotics to treat campylobacteriosis. Nevertheless, search on medicinal plants should be thorough in Sub-Saharan African region.

## Figures and Tables

**Table 1:** Detailed information about the *in vitro* studies of plants from Sub-Saharan Africa tested for activity against *Campylobacter* spp.

Plant	Family	Country of plant origin	Part	Type of extract (compound)	Inhibition zone (mm)	MIC (*μ*g/ml)	MBC (*μ*g/ml)	References
*Acacia caffra* (Thunb.) Willd.	Fabaceae	South Africa	Bark	70% Act	—	6250 (Cj)	—	[35]
				Aq	—	3130 (Cj)	—	
			Leaves	70% Act	—	6250 (Cj)	—	
				Aq	—	6250 (Cj)	—	
*Acacia nilotica* (L.) Delile	Fabaceae	Nigeria	Leaves	EtOH	6^a^(Cj, Cc, Cl)	80000 (Cj, Cc, Cl)	—	[24]
				Aq	2^a^(Cj, Cc, Cl)	250000 (Cj, Cc, Cl)	—	
*Albertisia villosa* (Exell) Forman	Menispermaceae	Democratic Republic of Congo (DRC)	Root	Et_2_O	—	>1000 (Cj, Cc)	—	[29]
				MeOH	—	62.5 (Cj, Cc)	—	
				Aq	—	62.5 (Cj, Cc)	—	
*Alepidea amatymbica* Eckl. & Zeyh.	Apiaceae	South Africa	Whole	70% Act	—	6250 (Cj)	—	[35]
				Aq	—	1560 (Cj)	—	
*Annickia chlorantha* (Oliv.) Setten & Maas	Annonaceae	Cameroon	Stem bark	Aq	30^b^(Cj/c)	390 (Cj/c)	1560 (Cj/c)	[31]
*Annona* sp.	Annonaceae	South Africa	Fruit	Act	—	750 (Csp)	—	[33]
				EA	—	6000 (Csp)	—	
				Hex	—	350 (Csp)	—	
*Bauhinia galpinii* N.E.Br.	Fabaceae	South Africa	Bark	Act	—	750 (Csp)	—	[33]
*Bridelia micrantha* (Hochst.) Baill.	Phyllanthaceae	South Africa	Bark	MeOH	—	3000 (Csp)	—	[33]
			Root	MeOH	—	3000 (Csp)	—	
			Seed	Act	—	750 (Csp)	—	
*Carissa spinarum* L.	Apocynaceae	South Africa	Leaves	Act	—	1500 (Csp)	—	[33]
*Cassine transvaalensis* (Burtt Davy) Codd	Celastraceae	South Africa	Root	MeOH	—	750 (Csp)	—	[33]
*Cissampelos torulosa* E.Mey. ex Harv. & Sond.	Menispermaceae	South Africa	Leaves	MeOH	—	750 (Csp)	—	[33]
*Cissus rubiginosa* (Welw. ex Baker) Planch.	Vitaceae	DRC	Stem bark	Et_2_O	—	>1000 (Cj, Cc)	—	[29]
				MeOH	—	1000 (Cj), 500 (Cc)	—	
				Aq	—	250 (Cj) 125 (Cc)	—	
*Combretum kraussii* Hochst.	Combretaceae	South Africa	Leaves and bark	70% Act	—	12500 (Cj)	—	[35]
				Aq	—	>12500 (Cj)	—	
*Combretum woodii* Dümmer	Combretaceae	South Africa	Leaves	Act and EtOH	—	40 (Cj)	—	[26]
*Croton mubango* Müll.Arg.	Euphorbiaceae	DRC	Stem bark	Et_2_O	—	>1000 (Cj, Cc)	—	[29]
				MeOH	—	1000 (Cj, Cc)	—	
				Aq	—	1000 (Cj), >1000 (Cc)	—	
*Cryptolepis sanguinolenta* (Lindl.) Schltr.	Apocynaceae	Guinea-Bissau (plant bought there)	Root	EtOH (cryptolepine)	—	6.25^c^, 12.5^d^(Cj) 12.5^c^, 25^d^(Cc)	—	[30]
			Root	EtOH	—	25^c^, 100^d^(Cj, Cc)	—	
			Root	Aq	—	200^c^, 400^d^(Cj) 400^c^, >400^d^(Cc)	—	
			Root	EtOH	29 (Cj) 34 (Cc)	—	—	[27]
*Elephantorrhiza burkei* Benth.	Fabaceae	South Africa	Root	70% Act	—	390 (Cj)	—	[35]
				Aq	—	3130 (Cj)	—	
*Eriosema cordatum* E.Mey.	Fabaceae	South Africa	Root	70% Act	—	>12500 (Cj)	—	[35]
				Aq	—	>12500 (Cj)	—	
*Ficus natalensis* Hochst.	Moraceae	Nigeria	NR	Hex	R (Cj)	—	—	[36]
				EA	R (Cj)	—	—	
				MeOH	R (Cj)	—	—	
*Ficus sycomorus* L.	Moraceae	South Africa	Bark	Act	—	350 (Csp)	—	[33]
*Gardenia ternifolia* Schumach. & Thonn. subsp., jovis-tonantis var. *goetzei* (Stapf and Hutch.) Verdc.	Rubiaceae	Guinea-Bissau	Root	Aq	9 (Cj) 10 (Cc)	—	—	[27]
*Gunnera perpensa* L.	Gunneraceae	South Africa	Rhizome	70% Act	—	12500 (Cj)	—	[35]
				Aq	—	>12500 (Cj)	—	
			Leaves	70% Act	—	780 (Cj)	—	
*Gymnanthemum glaberrimum* (Welw. ex O.Hoffm.) H.Rob.	Asteraceae	Nigeria	Leaves	Hex	0 (Cf)	—	—	[28]
				CF	0 (Cf)	—	—	
				EA	0 (Cf)	—	—	
				BuOH	0 (Cf)	—	—	
*Hypoxis obtusa* Burch. ex Ker Gawl.	Hypoxidaceae	South Africa	Corm	70% Act	—	1560 (Cj)	—	[35]
				Aq	—	780 (Cj)	—	
*Lippia javanica* (Burm.f.) Spreng.	Verbenaceae	South Africa	Leaves	MeOH	—	90 (Csp)	—	[33]
			Leaves	Act	—	90 (Csp)	—	
			Leaves	Essential oil	—	1500 (Csp)	—	
*Mimusops obovata* Sond.	Sapotaceae	South Africa	Bark	70% Act	—	12500 (Cj)	—	[35]
				Aq	—	>12500 (Cj)	—	
*Momordica balsamina* L.	Cucurbitaceae	South Africa	Leaves	Act	—	350 (Csp)	—	[33]
			Leaves	Hex	—	3000 (Csp)	—	
*Mucuna coriacea* Baker	Fabaceae	South Africa	Root	MeOH	—	3000 (Csp)	—	[33]
*Peltophorum africanum* Sond.	Fabaceae	South Africa	Bark	MeOH	—	1500 (Csp)	—	[33]
			Bark	70% Act	—	780 (Cj)	—	[35]
				Aq	—	3125 (Cj)	—	
			Leaves	70% Act	—	390 (Cj)	—	
				Aq	—	>12500 (Cj)	—	
				Aq	—	>12500 (Cj)	—	
*Pouzolzia mixta* Solms	Urticaceae	South Africa	Root	MeOH	—	750 (Csp)	—	[33]
			Stem	MeOH	—	750 (Csp)	—	
			Leaves	MeOH	—	3000 (Csp)	—	
			Root	Aq	—	6000 (Csp)	—	
*Pterocarpus angolensis* DC.	Fabaceae	South Africa	Bark	MeOH	—	1500 (Csp)	—	[33]
			Bark	Act	—	750 (Csp)	—	
			Bark	DCM	—	750 (Csp)	—	
			Bark	EA	—	90 (Csp)	—	
			Bark	Hex	—	1500 (Csp)	—	
			Bark	CF	—	750 (Csp)	—	
*Pterocarpus santalinoides* DC.	Fabaceae	Nigeria	Leaves	EA (friedelan-3-one)	R (Cj)	—	—	[32]
*Quassia africana* (Baill.) Baill.	Simaroubaceae	DRC	Root bark	Et_2_O	—	>1000 (Cj, Cc)	—	[29]
				MeOH	—	1000 (Cj, Cc)	—	
				Aq	—	1000 (Cj), >1000 (Cc)	—	
*Rhoicissus tridentata* (L.f.) Wild and R.B. Drumm.	Vitaceae	South Africa	Tuber	Act	—	750 (Csp)	—	[33]
			Fruit	MeOH	—	350 (Csp)	—	
			Fruit	Act	—	180 (Csp)	—	
*Rourea obliquifoliolata* Gilg	Connaraceae	DRC	Root	Et_2_O	—	500 (Cj), >1000 (Cc)	—	[29]
				MeOH	—	500 (Cj) 125 (Cc)	—	
				Aq	—	125 (Cj), 250 (Cc)	—	
*Searsia chirindensis* (Baker f.) Moffett	Anacardiaceae	South Africa	Leaves	Crude (MeOH)	—	780 (Cj)	—	[34]
				DCM	—	6250 (Cj)	—	
				EA	—	780 (Cj)	—	
				BuOH	—	3125 (Cj)	—	
				EA (methyl gallate)	—	60 (Cj)	—	
				EA (myricetin-3-O-arabinopyranoside)	—	130 (Cj)	—	
				EA (myricetin-3-O-rhamnoside)	—	130 (Cj)	—	
				EA (kaempferol-3-O- rhamnoside)	—	130 (Cj)	—	
				EA (quercetin-3-O-arabinofuranoside)	—	250 (Cj)	—	
Senna podocarpa (Guill. & Perr.) Lock	Fabaceae	Guinea-Bissau	Root	EtOH	8 (Cj) 9 (Cc)	—	—	[27]
			Leaves	EtOH	10 (Cj) 14 (Cc)	—	—	[27]
*Sida spinosa* L.	Malvaceae	South Africa	Leaves	Act	—	750 (Csp)	—	[33]
*Stephania abyssinica* (Quart.-Dill. & A.Rich.) Walp.	Menispermaceae	South Africa	Root	70% Act	—	6250 (Cj)	—	[35]
				Aq	—	>12500 (Cj)	—	
*Syzygium cordatum* Hochst. ex Krauss	Myrtaceae	South Africa	Bark	MeOH	—	350 (Csp)	—	[33]
			Bark	Act	—	180 (Csp)	—	
			Leaves	MeOH	—	3000 (Csp)	—	
			Leaves	Act	—	750 (Csp)	—	
*Tapinanthus globiferus* (A.Rich.) Tiegh.	Loranthaceae	Nigeria	Stem	Hex	R (Cj)	—	—	[25]
				EA	R (Cj)	—	—	
				MeOH	R (Cj)	—	—	
*Terminalia macroptera* Guill. & Perr.	Combretaceae	Guinea-Bissau	Root	EtOH	—	25^c^, 50^d^(Csp)	—	[23]
*Watsonia densiflora* Baker	Iridaceae	South Africa	Bulb	70% Act	—	12500 (Cj)	—	[35]
				Aq	—	>12500 (Cj)	—	
*Vitex doniana* Sweet	Lamiaceae	Nigeria	Bark	EtOH	1^a^(Cj, Cc, Cl)	NIE (Cj, Cc, Cl)	—	[24]
				Aq	0^a^(Cj, Cc, Cl)	NIE (Cj, Cc, Cl)	—	
*Vernonia amygdalina* Delile	Asteraceae	DRC	Leaves	Et_2_O	—	>1000 (Cj, Cc)	—	[29]
				MeOH	—	1000 (Cj, Cc)	—	
				Aq	—	>1000 (Cj, Cc)	—	
*Ximenia caffra* Sond.	Olacaceae	South Africa	Bark	Act	—	350 (Csp)	—	[33]
			Leaves	Act	—	750 (Csp)	—	
*Zornia milneana* Mohlenbr.	Fabaceae	South Africa	Whole	MeOH	—	180 (Csp)	—	[33]
			Whole	Act	—	180 (Csp)	—	

^a^Zone of inhibition at 2 mg of the extract; ^b^zone of inhibition at 12 mg of the extract; ^c^MIC 50%; ^d^MIC 90%; Csp: *Campylobacter* spp., Cc: *C. coli*, Cj: *C. jejuni*, Cj/c: *C. jejuni*/*C. coli*, Cl: *C. lari*, Cf: *C. fetus*, EtOH: ethanol; MeOH: methanol, Aq: aqueous, Et_2_O: diethyl ether, DCM: dichloromethane, EA: ethyl acetate, BuOH: butanol, Act: acetone, Hex: hexane, R: resistant, CF: chloroform, NIE: no inhibitory effect, NR: not reported.

## Supplementary Materials

Supplementary Materials The supplementary file is the search strategy in the databases (Pubmed, Science Direct, Google Scholar, and AJOL) for the systematic review.
