# Supplementary material for: A Systematic Review of In Vitro Activity of Medicinal Plants from Sub-Saharan Africa against Campylobacter spp
Source: Evid Based Complement Alternat Med. 2020 May 15;2020:9485364. doi: 10.1155/2020/9485364 (PMC7245682; doi:10.1155/2020/9485364)
Supplement: Supplementary Materials — The supplementary file is the search strategy in the databases (Pubmed, Science Direct, Google Scholar, and AJOL) for the systematic review. [file 9485364.f1.docx]

**A systematic review of *in vitro* activity of medicinal plants from Sub-Saharan Africa against *Campylobacter* spp.**

| **Searh Strategy** |
| --- |

12 March 2020

**Database: PubMed**

| **#** | **Research question** | **Records found** |
| --- | --- | --- |
| **#1** | Campylobacteriosis[Mesh] OR Campylobacter[Mesh] AND Medicinal plant[Mesh] OR campylob*[tiab] | 16721 |
| **#2** | Africa South of the Sahara[Mesh] OR Africa south of the Sahara[tiab] OR sub-Saharan[tiab] OR subSaharan[tiab] OR Angola[tiab] OR Benin[tiab] OR Botswana[tiab] OR British Indian Ocean Territory[tiab] OR Burkina Faso[tiab] OR Burundi[tiab] OR Cape Verde[tiab] OR Cameroon[tiab] OR Central African Republic[tiab] OR Chad[tiab] OR Comoros[tiab] OR Congo[tiab] OR Cote d’Ivoire[tiab] OR Democratic Republic of the Congo[tiab] OR DRC[tiab] OR Zaire[tiab] OR Djibouti[tiab] OR Equatorial Guinea[tiab] OR Eritrea[tiab] OR Eswatini[tiab] OR Swaziland[tiab] OR Ethiopia[tiab] OR French Southern Territories[tiab] OR Gabon[tiab] OR Gambia[tiab] OR Ghana[tiab] OR (Guinea[tiab] NOT guinea pig*[tiab]) OR Guinea-Bissau[tiab] OR Kenya[tiab] OR Lesotho[tiab] OR Liberia[tiab] OR Madagascar[tiab] OR Malawi[tiab] OR Mali[tiab] OR Mauritania[tiab] OR Mauritius[tiab] OR Mayotte[tiab] OR Mozambique[tiab] OR Namibia[tiab] OR Niger[tiab] OR Nigeria[tiab] OR Reunion[tiab] OR Rwanda[tiab] OR Saint Helena[tiab] OR Sao Tome and Principe[tiab] OR Senegal[tiab] OR Seychelles[tiab] OR Sierra Leone[tiab] OR Somalia[tiab] OR South Africa[tiab] OR South Sudan[tiab] OR Togo[tiab] OR Uganda[tiab] OR Tanzania[tiab] OR United Republic of Tanzania[tiab] OR Zambia[tiab] OR Zimbabwe[tiab] OR Central Africa*[tiab] OR Eastern Africa*[tiab] OR East Africa*[tiab] OR Southern Africa*[tiab] OR Western Africa*[tiab] OR West Africa*[tiab] | 300232 |
| **#3** | #1 AND #2 | **379** |

**Database: Science Direct**

| ***#*** | ***Searches*** | ***Results*** |
| --- | --- | --- |
| **1** | Find articles with these terms: Campylobacter OR Campylobacteriosis AND medicinal plant. Advanced search, Title, abstract, keywords: "Africa south of the Sahara" OR sub-Saharan OR subSaharan OR Angola OR Benin OR Botswana OR "British Indian Ocean Territory" | 34 |
| **2** | Find articles with these terms: Campylobacter OR Campylobacteriosis AND medicinal plant. Advanced search, Title, abstract, keywords: "Burkina Faso" OR Burundi OR "Cape Verde" OR Cameroon OR "Central African Republic" OR Chad OR Comoros OR Congo OR "Cote d’Ivoire" | 25 |
| **3** | Find articles with these terms: Campylobacter OR Campylobacteriosis AND medicinal plant. Advanced search, Title, abstract, keywords: "Democratic Republic of the Congo" OR DRC Zaire OR Djibouti OR "Equatorial Guinea" OR Eritrea OR Eswatini OR Swaziland OR Ethiopia | 14 |
| **4** | Find articles with these terms: Campylobacter OR Campylobacteriosis AND medicinal plant. Advanced search, Title, abstract, keywords: "French Southern Territories" OR Gabon Gambia OR Ghana OR (Guinea NOT guinea pig) OR Guinea-Bissau OR Kenya OR Lesotho OR Liberia | 36 |
| **5** | Find articles with these terms: Campylobacter OR Campylobacteriosis AND medicinal plant. Advanced search, Title, abstract, keywords: Madagascar OR Malawi OR Mali OR Mauritania OR Mauritius OR Mayotte OR Mozambique OR Namibia OR Niger | 26 |
| **6** | Find articles with these terms: Campylobacter OR Campylobacteriosis AND medicinal plant. Advanced search, Title, abstract, keywords: Nigeria OR Reunion OR Rwanda OR "Saint Helena" OR "Sao Tome and Principe" OR Senegal OR Seychelles OR "Sierra Leone" OR Somalia | 41 |
| **7** | Find articles with these terms: Campylobacter OR Campylobacteriosis AND medicinal plant. Advanced search, Title, abstract, keywords: "South Africa" OR "South Sudan" OR Togo OR Uganda OR Tanzania OR "United Republic of Tanzania" OR Zambia OR Zimbabwe OR "Central Africa" | 78 |
| **8** | Find articles with these terms: Campylobacter OR Campylobacteriosis AND medicinal plant. Advanced search, Title, abstract, keywords: "Eastern Africa" OR "East Africa" OR "Southern Africa" OR "Western Africa" OR "West Africa" | 24 |
|  | Total | **278** |

**Article types: Research articles, Short communications*

**Database: Google Scholar**

*(without patents and citations)*

| ***#*** | ***Searches*** | ***Results*** |
| --- | --- | --- |
| **1** | allintitle: campylobacteriosis \| campylobacter \| “medicinal plant” allintitle:"Africa south of the Sahara" \| sub-Saharan \| subSaharan \| Angola \| Benin \| Botswana \| "British Indian Ocean Territory" \| "Burkina Faso" \| Burundi \| "Cape Verde" \| Cameroon | 37 |
| **2** | allintitle: campylobacteriosis \| campylobacter allintitle:"Central African Republic" \| Chad \| Comoros \| Congo \| "Cote d’Ivoire" \| "Democratic Republic of the Congo" \| DRC \| Zaire \| Djibouti \| "Equatorial Guinea" \| Eritrea \| Eswatini \| Swaziland | 0 |
| **3** | allintitle: campylobacteriosis \| campylobacter allintitle:Ethiopia \| "French Southern Territories" \| Gabon \| Gambia \| Ghana \| Guinea \| Guinea-Bissau \| Kenya \| Lesotho \| Liberia \| Madagascar \| Malawi \| Mali \| Mauritania \| Mauritius | 101 |
| **4** | allintitle: campylobacteriosis \| campylobacter allintitle: Mayotte \| Mozambique \| Namibia \| Niger \| Nigeria \| Reunion \| Rwanda \| "Saint Helena" \| "Sao Tome and Principe" \| Senegal \| Seychelles \| "Sierra Leone" \| Somalia | 151 |
| **5** | allintitle: campylobacteriosis \| campylobacter \| “medicinal plant” allintitle: "South Africa" \| "South Sudan" \| Togo \| Uganda \| Tanzania \| "United Republic of Tanzania" \| Zambia \| Zimbabwe \| "Central Africa" \| "Eastern Africa" \| "East Africa" | 71 |
| **6** | allintitle: campylobacteriosis \| campylobacter \| “medicinal plant” allintitle "Southern Africa" \| "Western Africa" \| "West Africa" | 10 |
|  | Total | **370** |

**Database: African Journals Online (AJOL)**

| ***#*** | ***Searches*** | ***Results*** |
| --- | --- | --- |
| **1** | campylob* | **38** |
